# Hyaluronic Acid-Functionalized Mesoporous Silica Nanoparticles Loading Simvastatin for Targeted Therapy of Atherosclerosis

**DOI:** 10.3390/pharmaceutics14061265

**Published:** 2022-06-14

**Authors:** Kechen Song, Zhuang Tang, Zhiling Song, Shiyu Meng, Xiaoxue Yang, Hui Guo, Yizhun Zhu, Xiaolin Wang

**Affiliations:** 1 School of Pharmacy and State Key Laboratory of Quality Research in Chinese Medicines, Macau University of Science and Technology, Taipa 999078, Macau, China; 1909853UCT20003@student.must.edu.mo (K.S.); 2009853JPP30003@student.must.edu.mo (Z.T.); 19098530CT20001@student.must.edu.mo (Z.S.); 20098535CT20001@student.must.edu.mo (S.M.); 20098533CT20002@student.must.edu.mo (X.Y.); yzzhu@must.edu.mo (Y.Z.); 2 School of Chemical Engineering and Technology, Sun Yat-Sen University, Zhuhai 519082, China

**Keywords:** atherosclerosis, mesoporous silica nanoparticles, simvastatin, hyaluronic acid, enzyme-responsive drug release

## Abstract

Atherosclerosis (AS) constitutes a major threat to human health, yet most current therapeutics are hindered in achieving desirable clinical outcomes by low bioavailability or serious side effects. Herein, we constructed an enzyme-responsive and macrophage-targeting drug delivery system (SIM@HA-MSN) which can potentially modulate the microenvironment of the atherosclerotic plaques characterized by excessive inflammation and overexpression of hyaluronidase (HAase) for precise AS treatment. More specifically, mesoporous silica nanoparticles (MSNs) were loaded with a lipid-lowering drug simvastatin (SIM) and further gated with hyaluronic acid (HA) coating, which endowed the nanosystem with HAase responsiveness and targetability to inflammatory macrophages. Our results showed that a high loading efficiency (>20%) and excellent enzyme-responsive release of SIM were simultaneously achieved for the first time by silica-based nanocarriers through formulation optimizations. Moreover, in vitro experiments confirmed that SIM@HA-MSN possessed robust targeting, anti-inflammatory, and anti-foaming effects, along with low cytotoxicity and excellent hemocompatibility. In addition, preliminary animal experiments demonstrated the as-established nanosystem had a long plasma-retention time and good biocompatibility in vivo. Taken together, SIM@HA-MSN with HA playing triple roles including gatekeeping, lesion-targeting, and long-circulating holds great potential for the management of atherosclerosis.

## 1. Introduction

Atherosclerosis (AS) is the leading cause of a variety of cardiovascular diseases (CVDs) and accounts for one third of global mortalities [1]. AS is a chronic inflammatory disease characterized by plaque formation due to lipid retention in the medium to large artery wall and infiltration of monocytes, the progression and rupture of which can finally lead to lethal events such as myocardial infarction and ischemic stroke [2]. Traditional drug therapies including lipid-lowering drugs (e.g., statins, fibrates), platelet aggregation inhibitors, antihypertensive drugs, and anti-diabetic drugs (e.g., thiazolidinediones) generally suffer from low therapeutic efficacy and severe side effects, necessitating the development of novel drug carriers for precise AS treatment [3]. Consequently, a variety of organic or inorganic nanoparticles have emerged to combat AS by engineering nanoagents with versatile functionalization [4,5].

Amongst the nanoagents available for AS treatment, mesoporous silica nanoparticles (MSN) have attracted extensive attention owing to their remarkable properties including highly ordered channels, large surface areas, high pore volume, versatile surface functionality, and good biocompatibility [6]. They can achieve high loading of a broad range of therapeutic payloads either in the interior or the exterior surface while releasing the cargo in a controlled manner. Moreover, the drug release can be further manipulated by modification with a stimuli-responsive “gatekeeper”, which can be removed to discharge the sealed cargo under specific environmental stimuli, including pH [7], ROS [8], enzymes [9], glucose [10], temperature [11], light [12] ultrasound [13], etc. Moreover, the particle surface can be conjugated with a variety of targeting moieties to achieve precise drug delivery, such as antibodies [14], peptides [15], and polysaccharide [16].

In the initiation and progression of AS, macrophages play a pivotal role and have become a potential target for AS therapy [17]. They ingest mainly modified lipoprotein (e.g., ox-LDL) to develop into foam cells, which further induce a cascade of inflammatory responses that promote more lipoprotein retention, extracellular matrix (ECM) modification, and chronic inflammation [18]. Therefore, it is of high value to develop a smart drug delivery system to modulate macrophage behaviors in plaques [19,20]. To this aim, hyaluronic acid (HA), a disaccharide unit glycosaminoglycan, was chosen as the functional modality on the MSN surface for serval reasons. First of all, HA is a key component in the ECM of animals and thus has excellent biocompatibility [21]. In addition, it has been well documented that HA can recognize and bind to CD44 receptors expressed on inflammatory macrophages [22]. Moreover, the concentration of hyaluronidase (HAase) in atherosclerotic plaques is significantly higher than that in normal tissues [23]. Therefore, the HA layer can maintain the drug in a well-sealed manner in the pores of MSN and responsively liberate the cargos to the lesion readily upon degradation by HAase. Further, HA can serve as a molecular brush to prevent nanoparticles from clearance by the reticuloendothelial system (RES), which plays an important role in host defense, thus prolonging the action time of nanoparticles in the body [24]. Last but not least, HA exhibited plural biological properties including anti-inflammation, which can contribute to the inflammation resolution at the plaques [25].

Simvastatin (SIM) is among the most often prescribed lipid-lowering medications [26], and also exhibits anti-inflammatory and antioxidant effects, favoring AS treatment [27]. However, it has low bioavailability due to the poor water solubility (0.0004 mg/mL) [28]. In addition, the side effects such as myopathy and liver damage also limit its clinical application [29]. To enhance its bioavailability while averting the side effects, we constructed an enzyme-responsive and macrophage-targeting nanosystem (SIM@HA-MSN). More specifically, MSNs serve as drug reservoirs for SIM deposits. Subsequently, polyethyleneimine (PEI), a cationic polymer, was absorbed on the particle surface through electrostatic interaction and provided plenty of amine groups for HA conjugation via amide bond. To the best of our knowledge, silica-based nanocarriers have so far failed to deliver SIM in a targeted and controlled manner. Thus, we hypothesize that SIM@HA-MSN could deliver SIM in response to enzyme stimuli in the plaque microenvironment to downregulate inflammation and inhibit foam cell formation to favor the regression of atherosclerosis. Even though MSNs are widely investigated drug carriers, they are rarely reported for CVD treatment due to concerns arising over toxicity [30,31]. Hence, the biosafety of SIM@HA-MSN in vitro and in vivo was also evaluated in our study.

## 2. Materials and Methods

### 2.1. Materials

All reagents were of analytical or HPLC grade and used without any further purification. Tetraethyl orthosilicate (TEOS), cetyl trimethyl ammonium bromide (CTAB), branched polyethyleneimine (PEI) (Mw = 10 kDa), (3-aminopropyl) triethoxysilane (APTES), and sodium dodecyl sulfate (SDS) were purchased from Macklin. Co., Ltd. Fluorescein isothiocyanate isomer (FITC), Simvastatin (SIM), hyaluronidase (HAase), N-hydroxy succinimide (NHS), 1-(3-Dimethylaminopropyl)-3-ethylcarbodiimide hydrochloride (EDC), Resazurin, 4,6-diamidino-2-phenylindole (DAPI), and lipopolysaccharide (LPS) were purchased from Sigma-Aldrich Inc. (St. Louis, MO, USA). Sodium hyaluronate (HA) (Mw = 200~400 kDa) was purchased from Bloomage Biotechnology Co., Ltd. (Jinan, China). Oxidized low-density lipoprotein (ox-LDL) was purchased from Yiyuan Biotechnology Co., Ltd. (Bozhou, China). Phosphate buffer tablets were purchased from Amresco Inc. (Cleveland, OH, USA). The mouse ELISA kits to measure the secretion of tumor necrosis factor-α (TNF-α) and interleukin-6 (IL-6) levels were purchased from Beyotime Biotechnology Co., Ltd. (Shanghai, China).

### 2.2. Synthesis of MSN

MSNs were synthesized according to the literature with minor modification [32]. Briefly, 0.4 g CTAB was dissolved in 200 mL ultrapure water containing 1.44 mL NaOH aqueous solution (2 M) and heated to 80 °C under stirring. Subsequently, 0.4 mL anhydrous ethanol was added and the solution was stirred for another 5 min. Then, 2 mL TEOS solution was added dropwise, and the reaction was maintained for 2 h at 80 °C. The product was centrifuged (10,200 rpm, 15 min) and washed three times with ethanol and deionized water to remove the residual impurities. To remove the CTAB template, the product was dried overnight and calcined at 550 °C for 5 h. Finally, white powders were obtained and denoted as MSNs.

### 2.3. Synthesis of HA Modified MSN (HA-MSN)

In 4 mL phosphate-buffered saline (PBS, 10 mM, pH = 7.4), 30 mg MSNs was dispersed, followed by the addition of 100 μL PEI solution (75 mg/mL). The mixtures were stirred for 30 min at room temperature. Then, the product was centrifuged (10,200 rpm, 15 min), and washed with ultra-pure water 3 times to obtain PEI-MSN. Then, 15 mg HA, 40 mg NHS, and 30 mg EDC were dissolved in 10 mL distilled water and stirred for 1 h to activate the carboxyl groups in hyaluronic acid. Subsequently, PEI-MSN was added to the mixture, which was then stirred for another 24 h at room temperature. Finally, HA-MSN was obtained after centrifugation, washing, and overnight lyophilization.

### 2.4. Synthesis of FITC-Labeled MSN and HA-MSN

To obtain FITC-labeled MSN, amino-conjugated nanoparticles (NH_2_-MSN) were synthesized according to the literature [33]. Briefly, 100 mg MSN was dispersed in 50 mL methanol after 30 min of ultrasound treatment. After being gently heated to 40 °C, 0.4 mL APTES was slowly added to the solution, which was then stirred overnight. The mixture was then centrifuged, washed with methanol 5 times, and dried in a vacuum overnight to obtain NH_2_-MSN. Subsequently, 30 mg NH_2_-MSN and HA-MSN powder were dispersed in 2 mL distilled water, and 1 mL FITC ethanol solution (1 mg/mL) was added, respectively. After overnight stirring in the dark, the products were collected by centrifugation and washed with ethanol three times to remove free FITC. Finally, FITC-MSN and FITC-HA-MSN were obtained by drying overnight in a vacuum desiccator at 40 °C.

### 2.5. Physicochemical Characterizations

The size and morphology of particles were observed with a transmission electron microscope (TEM, JEM 2100F, JEOL, Tokyo, Japan). Brunauer–Emmett–Teller (BET) measurement and Barrett–Joyner–Halenda (BJH, ASAP2460, Micromeritics Inc., Norcross, GA, USA) analysis were employed to determine the surface area and the pore size distribution of different particles. The particle size and zeta potential were measured using Malvern ZetaSizer Nano ZS90 (Malvern Instruments, Malvern, UK). The X-ray diffraction patterns (XRD, UltimaIV, Rigaku, Tokyo, Japan) of SIM, MSN, and SIM@HA-MSN were analyzed with X’Pert PRO MPD (Panalytical, Amsterdam, The Netherlands). The thermal properties were determined by differential scanning calorimetry (DSC, DSC250, TA Instruments, New Castle, DE, USA). Chemical modification was proved by Fourier transform infrared spectroscopy (FT-IR, Nicolet 5700 FT-IR Spectrometer, Thermo Fisher Scientific, Massachusetts, USA). Thermogravimetry analysis (TGA, TA Instruments, New Castle, DE, USA) was used to calculate the amount of polymer conjugation by heating the samples at a rate of 10 °C/min from RT to 1000 °C in air flow.

### 2.6. Drug Loading

SIM was successfully loaded in MSNs by solvent impregnation. Briefly, 30 mg of MSN and 15 mg of SIM were mixed in 2 mL of dichloromethane and stirred for 24 h. Then, the nanoparticle was collected by centrifugation and dried at room temperature for 12 h in a vacuum oven to obtain SIM@MSN. Afterward, PEI and HA were modified following the same procedure as introduced in Section 2.4 to finally obtain SIM@HA-MSN. To evaluate the drug-loading capacity of SIM@ HA-MSN, supernatant after each centrifugation was collected to detect free SIM by UV-2450 (UV-vis spectrophotometer, Shimadzu) at a wavelength of 238 nm. Drug-loading efficiency (LE) and encapsulation efficiency (EE) were calculated by the following equations.

(1) $$\mathrm{LE}\;\left(\%\right)=\frac{\mathrm{Weight}\;\mathrm{of}\;\mathrm{total}\;\mathrm{drug}-\mathrm{Weight}\;\mathrm{of}\;\mathrm{free}\;\mathrm{drug}\;}{\mathrm{Weight}\;\mathrm{of}\;\mathrm{nanoparticles}}\times 100\%$$

(2) $$\mathrm{EE}\;\left(\%\right)=\frac{\mathrm{Weight}\;\mathrm{of}\;\mathrm{total}\;\mathrm{drug}-\mathrm{Weight}\;\mathrm{of}\;\mathrm{free}\;\mathrm{drug}}{\mathrm{Weight}\;\mathrm{of}\;\mathrm{total}\;\mathrm{drug}}\times 100\%$$

### 2.7. In Vitro Drug Release

The release profile of SIM from SIM@HA-MSN and SIM@MSN was monitored by the direct diffusion method as SIM has too strong an affinity to the dialysis bags to diffuse out. Briefly, 10 mg nanoparticles were dispersed homogeneously in 40 mL PBS (10 mM, containing 0.2% SDS, pH = 7.4). To characterize the enzyme responsiveness, hyaluronidase was added to reach a final concentration of 100 U/mL. The samples were shaken at 100 rpm at 37 °C. At designated time intervals (1 min, 5 min, 10 min, 0.5 h, 1 h, 2 h, 4 h, 24 h, 48 h for free SIM and 10 min, 1 h, 2 h, 6 h, 21 h, 48 h for SIM-loaded nanocarriers), the samples were centrifuged, 2 mL of buffer solution was collected, and the same volume of fresh buffer was replaced. Next, the amount of SIM released was determined with High Performance Liquid Chromatography (HPLC, Agilent 1200 series) at a wavelength of 238 nm with Agilent C18 column (4.6 mm × 250 mm, 5 µm). The mobile phase consisted of acetonitrile: 0.025 M sodium dihydrogen phosphate solution (76:24 V:V) pumped at a flow rate of 1.0 mL/min with an injection volume of 20 μL.

The cumulative release rate was calculated by the following equation.

(3) $$\mathrm{Cumulative}\;\mathrm{release}\;(\%)=\frac{M_{t}}{M_{0}}\;\times \;100\%$$

where *M_t_* was the amount of drug released at time *t* and *M*_0_ was the total amount of drug in the free form or loaded in the nanoparticles.

### 2.8. Cell Culture

Mouse macrophages (Raw264.7 cells) and Human Umbilical Vein Endothelial Cells (HUVECs) were purchased from the National Collection of Authenticated Cell Cultures of China. The cells were cultured in Dulbecco’s Modified Eagle medium (DMEM) supplemented with 10% fetal bovine serum (FBS), 100 U/mL penicillin, and 100 μg/mL streptomycin in a humidified incubator with 5% CO_2_ at 37 °C. The cells were harvested by scratching, centrifuged, and suspended in a fresh medium. The cell number was calculated using a standard trypan blue cell counting technique.

### 2.9. Cytotoxicity and Hemocompatibility Test

Cell viability was monitored using the Alamar Blue test. Raw264.7 cells and HUVECs were separately inoculated into 96-well plates at a density of 5 × 10^3^ cells per well at 37 °C in a moist atmosphere with 5% CO_2_. Then MSN, HA-MSN, and SIM@HA-MSN at different concentrations (0, 10, 50, 200, 400 μg/mL) were added to the 96-well plate separately. The culture medium was removed after co-incubation for 24 h, and replaced by 100 μL Alamar Blue solution (10% in cell culture medium) to incubate at 37 °C for another 4 h. Analysis was then performed on a microplate reader (SpectraMax ID5, Molecular Devices, Sunnyvale, CA, USA) at wavelengths of 570 nm and 600 nm. Cell viability was calculated and reported as a percentage of the control group (*n* = 3).

To conduct the hemolysis assay, whole blood from mice was collected using a heparin-containing tube and centrifuged at 2000 rpm for 10 min to obtain red blood cells (RBCs). Then, the pellets were washed three times and suspended in saline to obtain 2% RBC suspension. MSN and HA-MSN were suspended in normal saline and mixed with different volumes of RBC suspension to achieve a final concentration of 25, 50, 100, 200, 400, and 800 μg/mL. In addition, red blood cell samples mixed with normal saline and distilled water were used as negative and positive controls, respectively. The samples were incubated at 37 °C for 3 h and centrifuged at 1800 rpm for 10 min. Finally, the absorbance of the supernatant was measured at 540 nm. The hemolysis percentage of the RBCs was calculated as follows.

(4) $$\mathrm{Hemolysis}\;\left(\%\right)=\frac{A_{sample}-A_{negative}}{A_{positive}-A_{negative}}\times 100\%$$

### 2.10. Cellular Uptake of Nanoparticles

To investigate CD44-mediated cellular uptake, Raw264.7 cells were seeded in 24-well plates at a density of 5 × 10^4^ per well and incubated overnight at 37 °C in a moist atmosphere with 5% CO_2_. Then, 100 ng/mL LPS was added and incubated for 24 h. After PBS washing, FITC-modified HA-MSN and MSN were co-incubated with Raw264.7 cells separately for 2 h. The final concentration of each group was 50 μg/mL. After washing with PBS, the cells were fixed with 100 μL 4% PFA solution for 10 min in each well and stained with DAPI for 10 min. Finally, the cells were observed under a confocal laser scanning microscope (CLSM, Leica Stellaris, Frankfurt, Germany). In the competitive inhibition study, the blank medium was replaced with the HA-containing medium (10 mg/mL); then, FITC-labeled HA-MSN (50 mg/mL) was co-cultured with Raw264.7 cells, and the same treatment was performed as above. Other cell line HUVECs were adopted as a negative control and subjected to the same treatment as RAW 264.7 cells to validate the targetability of nanoparticles to inflammatory macrophages.

To investigate the intracellular drug release, a hydrophobic dye nile red (NR) was loaded into the HA-MSN following the same protocol for drug loading as described in Section 2.6, except that 30 mg MSN was mixed with 0.3 mg NR in the solvent and the final product was designated as NR@HA-MSN. Afterwards, Raw264.7 cells were seeded in 24-well plates at a density of 5 × 10^4^ per well and incubated overnight at 37 °C in a moist atmosphere with 5% CO_2_. Then 100 ng/mL LPS and 100 μg/mL ox-LDL were added separately and incubated for 24 h. Subsequently, the medium was removed and supplemented with fresh medium containing 50 μg/mL NR@HA-MSN and incubated for 1 h, 2 h, 4 h, and 8 h. After rinsing with PBS, the cells were fixed with 4% PFA for 10 min and stained with DAPI for 10 min. Finally, the cells were observed under a confocal laser scanning microscope (CLSM, Leica Stellaris, Frankfurt, Germany).

### 2.11. In Vitro Anti-Atherosclerosis Effects

#### 2.11.1. Anti-Inflammatory Effect on Macrophages

The aim was to detect the impacts of SIM@HA-MSN on the typical inflammatory factors secreted by Raw264.7 cells. Briefly, Raw264.7 grown in the logarithmic phase was inoculated into 24-well plates at a density of 10^5^/mL and incubated overnight in a 5% CO_2_ incubator at 37 °C. The positive control group was treated with 100 ng/mL LPS while the other groups were treated with free SIM, SIM@MSN, or SIM@HA-MSN at an equivalent SIM concentration of 10 µM together with 100 ng/mL LPS for 24 h. Cell supernatant of each group was collected by centrifugation, and the concentrations of TNF-α and IL-6 protein were detected with an ELISA kit following the manufacturer’s instructions. All experiments were repeated in triplicate.

#### 2.11.2. Inhibition of Foam Cell Formation

Raw264.7 cells were inoculated on 24-well plates at a density of 5 × 10^4^ per well and incubated overnight in a 5% CO_2_ incubator at 37 °C. The positive control group was treated with 100 μg/mL ox-LDL while the other groups were co-cultured with free SIM, SIM@MSN, or SIM@HA-MSN at the equivalent SIM concentration of 10 µM together with 100 μg/mL ox-LDL for 24 h. Afterward, the adherent macrophages in each group were washed with PBS 3 times, fixed with 4% PFA for 10 min, and stained with freshly filtered 0.3% oil red O for 15 min. Finally, the cells were placed in 60% isopropyl alcohol for 5 min and imaged with an optical microscope (Axiovert 135A, Zeiss, Jena, Germany).

### 2.12. Animals

Male C57BL/6 mice (6–8 weeks old) were purchased from SPF (Beijing) Biotechnology Co., Ltd. (Beijing, China). Animals were housed with *ad libitum* access to water and food. Before experiments, all mice were acclimatized for at least one week. All animal-related procedures were in line with the guidelines of the China Council on Animal Care and approved by the Animal Ethics Committee of the Macau University of Science and Technology.

### 2.13. Circulation Test In Vivo

FITC-MSN and FITC-HA-MSN were administrated through tail vein injection (200 μL, 5 mg/kg) separately, and then cheek blood was taken quickly at 1 min, 30 min, 1 h, 6 h, 12 h, 24 h, and 48 h. Afterward, 50 μL of blood was mixed with an equal volume of PBS each time, and then the blood was quantitatively transferred to a 96-well plate. The fluorescence intensity was measured by a microplate reader (SpectraMax Paradigm Multi-mode, Molecular Devices, Sunnyvale, CA, USA) at an excitation wavelength of 490 nm and an emission wavelength of 530 nm.

### 2.14. Biosafety Study

Mice were injected with SIM@MSN and SIM@HA-MSN intravenously at equivalent SIM dose at 15 mg/kg while the control group was injected with an equal volume of PBS. Injections were administrated every two or three days for four weeks. Subsequently, the blood was collected for serum biochemical analysis to study the impact of particles on liver and kidney function while H&E staining was performed on the sections of major organs (heart, liver, spleen, lung, and kidney).

### 2.15. Statistical Analysis

All data were displayed as mean ± SD. GraphPad Prism 8.0 software (GraphPad, San Diego, CA, USA) was used for data analysis. Statistical significance was assessed using one-way analysis of variance (ANOVA) followed by Tukey (compare all pairs of groups) or Dunnett (compare a control group with other groups) post-hoc test. Statistical significance was assessed at *p* < 0.05.

## 3. Results and Discussions

### 3.1. Preparation and Functionalization of MSN

**Figure 1:**
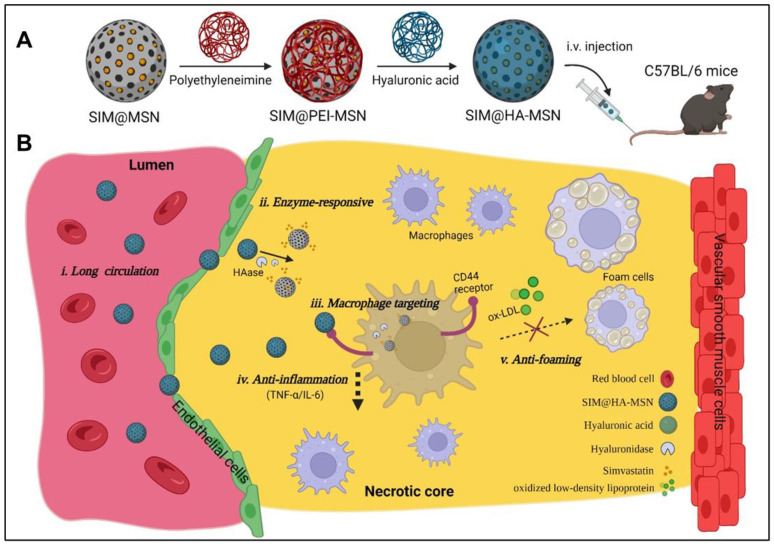
Schematic illustration of construction of SIM@HA-MSN for atherosclerosis management. (**A**) Scheme of fabrication process and administration route of SIM@HA-MSN. (**B**) Illustration of SIM@HA-MSN therapy which can potentially alleviate atherosclerosis through long circulating, enzyme-responsive drug release, macrophage targeting, and anti-inflammatory and anti-foaming effects. (Created by *Biorender*).

In this study, SIM@HA-MSN is designed for atherosclerosis management by targeting macrophages with high expression of the CD44 receptors in the plaque (Figure 1). To this end, MCM-41-type MSNs were first synthesized with CTAB as the templating agent and TEOS as the silica precursor [32]. After template removal, the particles with highly ordered mesoporous structures were further coated with positively charged polyethyleneimine (PEI) through electrostatic interaction [34,35]. PEI instead of amino-bearing silane (e.g., APTES) was chosen for surface modification owing to the facile and environment-friendly manipulation in aqueous media to enhance the drug loading efficiency. For example, our preliminary data showed that the drug loading capacity of APTES-modified MSN was extremely low, whether the conjugation was performed before (Figure S1) or after (not detectable) the drug loading.

In the subsequent step, the amine groups of PEI were covalently conjugated with the carboxyl (-COOH) of HA through EDC/NHS reaction to give rise to HA-decorated MSN (HA-MSN). In this conjugating process, covalent bonding instead of simple electrostatic adsorption was executed to avoid early disassociation of HA from the nanoparticles. Moreover, HA with high molecular weight was chosen for MSN functionalization due to superior biocompatibility and CD44 receptor targeting ability compared to the counterpart with low molecular weight [36,37,38].

### 3.2. Physicochemical Characterizations of MSN-Based Nanoparticles

**Figure 2:**
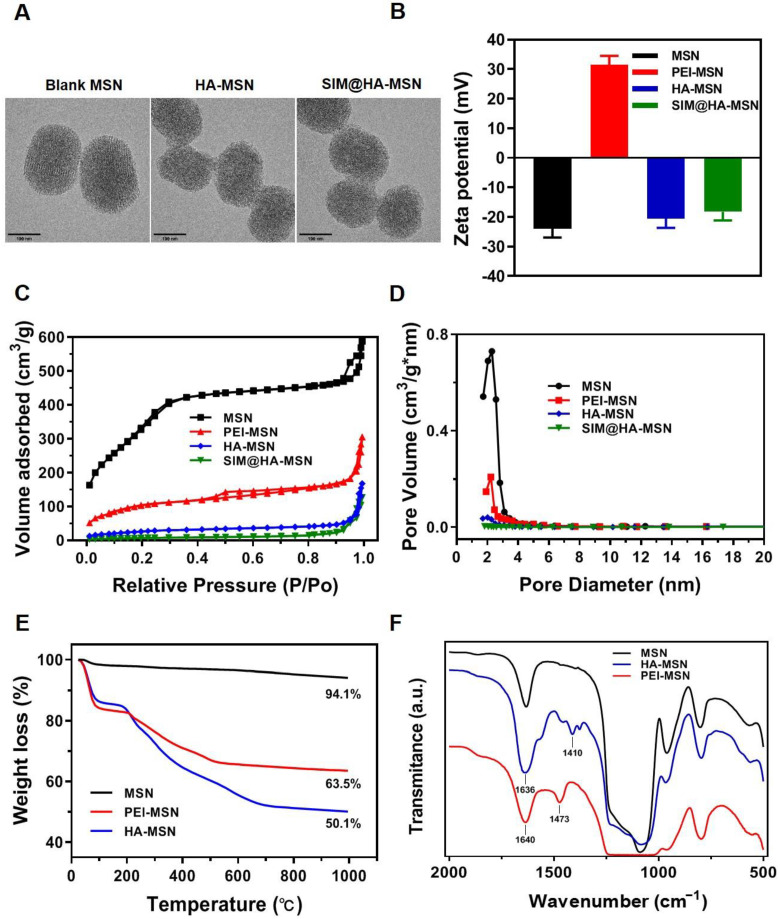
Physicochemical characterizations of MSN-based nanosystems. (**A**) TEM images (scale bar = 100 nm), (**B**) average zeta potentials, (**C**) N_2_ adsorption–desorption isotherms, and (**D**) the pore size distributions of bare MSN, PEI-MSN, HA-MSN, and SIM@HA-MSN. (**E**) TGA analysis and (**F**) FT-IR spectra of MSN, PEI-MSN, and HA-MSN.

TEM images showed that the as-obtained MSN was monodispersed with a size of ~200 nm and a typically ordered channel structure of MCM-41 (Figure 2A, left panel). Meanwhile, HA-MSNs and SIM@HA-MSN showed slightly larger sizes and the pores could not be observed due to the coverage of HA and/or encapsulation of SIM (Figure 2A, middle and right panel). In addition, the sizes of MSN, HA-MSN, and SIM@HA-MSN determined by DLS were 178.3 ± 5.2 nm, 185.4 ± 6.1 nm, and 189.1 ± 5.8 nm, respectively (Figure S2A), which were in accordance with TEM observation. SIM@HA-MSN demonstrated slightly larger size compared with HA-MSN, but it maintained the same size of ~190 nm in PBS over 1 week, suggesting desirable dispersity and stability for further applications (Figure S2B). Meanwhile, the zeta potential of MSN increased from −23.5 ± 2.4 mV to +31.6 ± 3.2 mV due to cationic PEI coating on the outer surface, which was then converted to be −20.2 ± 2.1 mV after HA conjugation (Figure 2B).

BJH and BET measurements were also executed to confirm the grafting of PEI and HA. The N_2_ adsorption–desorption isotherms of MSN exhibited type IV isotherm (Figure 2C), which is a typical characteristic of mesoporous silica [39]. The pore size distribution of MSNs was narrow, suggesting the uniformity of the pore channels (Figure 2D). As demonstrated in Figure 2C,D and Table 1, the sharply decreased BET surface area, pore volume, and pore size of PEI-MSN, HA-MSN compared with MSN indicated successful layer-by-layer modifications on the particle surface. Moreover, the further decreased surface and pore volume for SIM@HA-MSN indicated the inner surface was further occupied by drug molecules.

In the next step, the graft ratio of PEI and HA onto MSN was characterized with a TGA test. As shown in Figure 2E, MSNs demonstrated good thermostability as almost no degradable groups exist in the range of 25 to 1000 °C. In contrast, the PEI-MSN and HA-MSN experienced a striking weight loss at the temperature higher than 200 °C due to the degradation of organic components. After subtracting the interference of water molecules, the graft rates of PEI and HA were 19.3% and 17.4%, respectively, proving the successful grafting of the two polyelectrolytes.

In addition, the surface modifications were also confirmed by FT-IR (Figure 2F). The peaks at ~1089 cm^−1^ were assigned to the asymmetrical stretching of Si-O-Si bridges in MSNs. PEI-MSN showed the bending vibration (in-plane) of primary amine (-NH_2_) appeared at 1640 cm^−1^, and C-H bending vibration (in-plane) appeared at 1473 cm^−1^, indicating the successful modification of PEI on the surface of MSN. With the further modification of HA, C=O stretching vibration at 1636 cm^−1^ and C-N stretching vibration at 1410 cm^−1^ appeared, which were ascribed to the amide bond being formed between the -COOH of HA and -NH_2_ of PEI, which verified the success of HA conjugation.

### 3.3. Drug-Loading Properties

**Figure 3:**
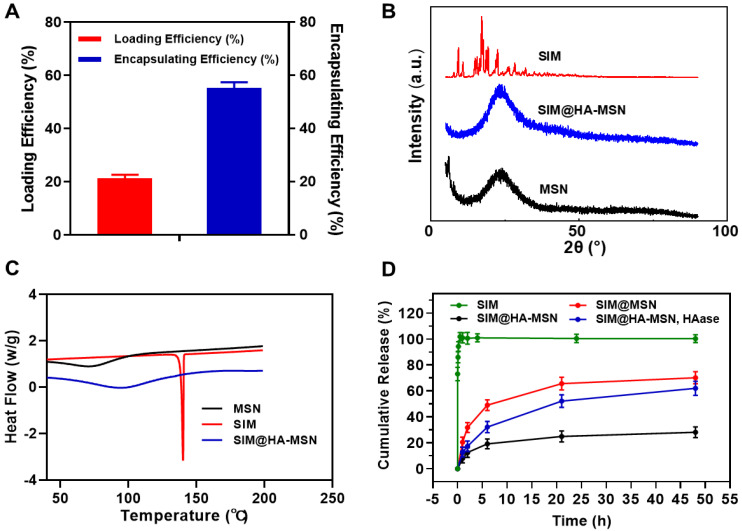
(**A**) Loading efficiency (LE%) and encapsulating efficiency (EE%) of SIM@HA-MSN (*n* = 3). (**B**) XRD spectrum and (**C**) DSC analysis of bare MSN, SIM, and SIM@HA-MSN. (**D**) In vitro drug release of free SIM, SIM@MSN, and SIM@HA-MSN in the presence or absence of HAase (100 U/mL) (*n* = 3).

SIM was previously reported to be loaded in porous silica nanoparticles with a solvent evaporation method using ethanol as solvent [40,41]. Despite that high drug loading of >30% was achieved, SIM was released instantly within 1 h, probably due to preferential adsorption on the outer surface during the drying process. In this study, we alternatively used the solvent impregnation method to avoid burst release while maintaining high drug loading by screening loading solvent, duration, and sequences (Figures S3–S5). Finally, the optimal loading conditions were determined to be co-incubation of bare MSN and SIM in dichloromethane with a loading duration of 24 h before polymer conjugation on the particle surface. As a result, the as-obtained formulation achieved LE% and EE% as high as 21.32 ± 1.31% and 55.21 ± 2.23%, respectively (Figure 3A). Hence, the drug-loading conditions should be optimized according to the physicochemical properties of specific drug molecules despite the high loading potential of MSN for a wide range of therapeutics.

XRD and DSC were further employed to investigate the interactions between SIM and nanoparticles (Figure 3B,C). In the XRD spectra, SIM exhibited a typical crystal profile, while MSN demonstrated an amorphous form (Figure 3B). The crystal peaks of the SIM were absent in that of SIM@HA-MSN, suggesting that SIM adopted an amorphous state inside the nanocarriers. In the DSC studies, free SIM possessed a single endothermic peak at 143 °C (Figure 3C), which was assigned to be the intrinsic melting point of SIM crystals [41]. After encapsulation into particles by solvent impregnation, the endothermic peak of SIM disappeared in the curve of SIM@HA-MSN, which again confirmed the amorphous state inside the mesopores.

### 3.4. In Vitro Drug Release Behaviors

The drug release kinetics of SIM@HA-MSN were studied to explore the drug-blocking efficiency in the physiological environment and enzyme-responsive release behavior due to surface modification (Figure 3D). The purpose of adding 0.2% SDS into the release media was to maintain the sink condition of hydrophobic SIM at 37 °C, which significantly increased the solubility of SIM from 0.4 μg/mL to 712 μg/mL in 10 mM PBS. Free SIM was instantly released into the medium within 1 h. In contrast, Sim@MSN manifested a sustained release of 70.11 ± 4.64% over 48 h, whereas the SIM@HA-MSN exhibited a significantly hindered release of 28.02 ± 4.11%, indicating SIM was embedded inside the mesopores and the release can be blocked effectively by HA coating [41].

To simulate a plaque environment with high levels of hyaluronidase (HAase) expression [23], HAase (100 U/mL) was added to the buffer. In contrast to low drug release in blank PBS, 62.24 ± 5.82% of the drug was released in 48 h due to the rapid rupture of the HA layer upon degradation by HAase. Overall, these results show that HA modification on SIM@HA-MSN can serve as a gatekeeper that can efficiently block the drug leakage at normal tissue but can be “switched on” in response to high-level HAase at the lesion site.

### 3.5. In Vitro Cytotoxicity and Hemocompatibility

**Figure 4:**
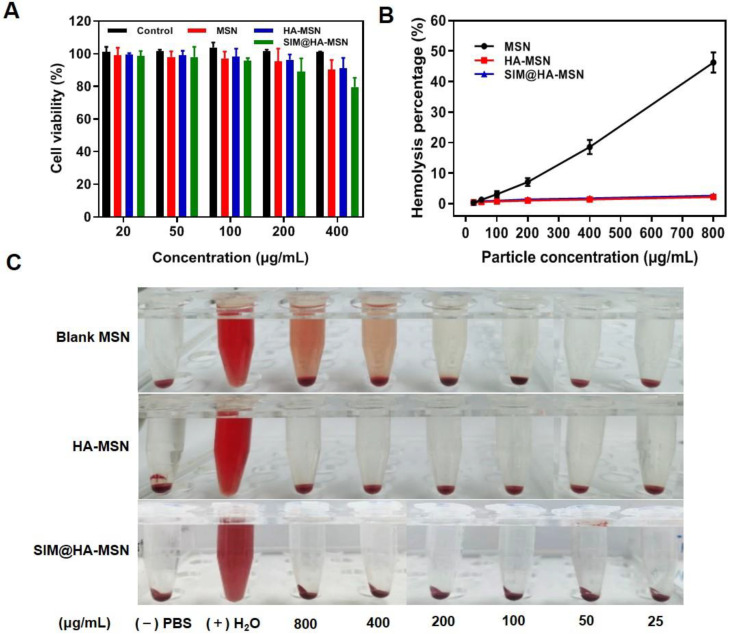
(**A**) Cytocompatibility of particles on Raw264.7 cells after co-incubation for 24 h (*n* = 3). (**B**) Hemolysis percentage and (**C**) images of RBCs incubated with MSN, HA-MSN, SIM@HA-MSN for 3 h. Distilled water and PBS were used as positive and negative controls, respectively (*n* = 3).

It was reported that MSNs with varied pore sizes, morphologies, and surface functionality affected the toxicity and their hemolytic activity [42]. We first investigated the cytotoxicity of MSN, HA-MSN, and SIM@HA-MSN on Raw264.7 cells and HUVECs by Alamar blue assay. After incubating the cells with HA-MSN and MSN at a dose of 20–400 μg/mL for 24 h, the survival rates of Raw 264.7 cells (Figure 4A) and HUVECs (Figure S6) were above 80% compared to the control group, suggesting that all the particles demonstrated good cytocompatibility. The decrease of cell viability at a high dose of SIM@HA-MSN probably resulted from the released SIM, which can inhibit the proliferation of macrophages to suppress plaque inflammation [43].

Subsequently, a hemolysis assay was conducted to evaluate the hemocompatibility of the particles (Figure 4B,C). Since the damage of RBCs could induce the release of hemoglobin, the hemolysis rate was determined by measuring the absorbance of hemoglobin in the supernatant at a wavelength of 576 nm. The results showed that the hemolysis rate of MSN was dose-dependent and increased to as high as 46.24 ± 3.92% at 800 μg/mL (Figure 4B) with notable hemolysis of RBCs (Figure 4C). In contrast, there was no obvious hemolysis of RBCs and negligible hemolytic rate for HA-MSN and SIM@HA-MSN (<5%). This result revealed that SIM@HA-MSN had superior hemocompatibility compared with bare MSN due to the shielding effect of HA, which was consistent with the previous report [44].

### 3.6. In Vitro Cellular Uptake

**Figure 5:**
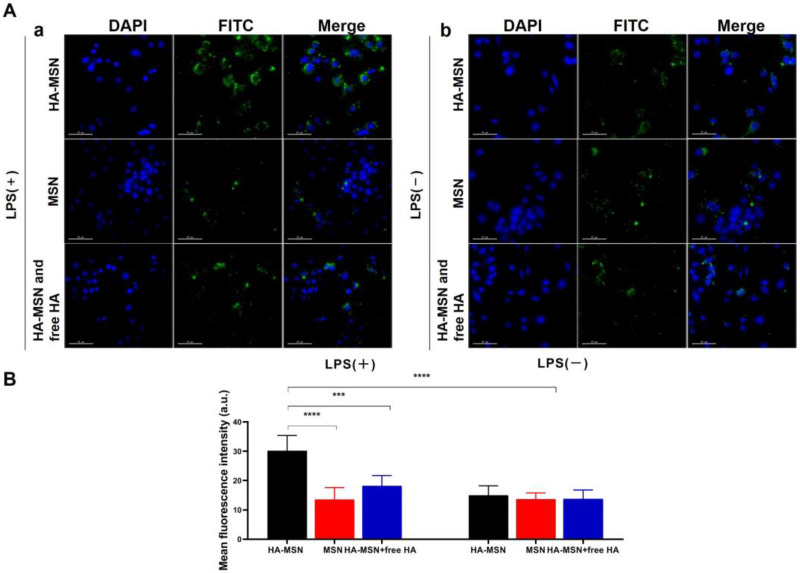
(**A**) Confocal laser microscope images of Raw264.7 cells treated with FITC-labeled HA-MSN, MSN, and free HA + FITC-HA-MSN for 2 h with (**a**) or without (**b**) LPS activation. (**B**) Mean fluorescence intensity of FITC-HA-MSN, FITC-MSN, and free HA + FITC-HA-MSN after cellular uptake (*n* = 3). Variance among groups was determined by one-way ANOVA with Tukey post-hoc test (*** *p* < 0.001, **** *p* < 0.0001).

To evaluate the selective targeting and internalization of inflammatory macrophages, FITC-labeled MSN and HA-MSN were co-cultured with LPS-activated (Figure 5Aa) or non-activated (Figure 5Ab) Raw264.7 cells for 2 h and cellular uptake was observed by confocal laser scanning microscope. Overall, the LPS-activated group manifested stronger fluorescence than the non-activated group owing to enhanced endocytosis of particles by inflammatory macrophages. As shown in Figure 5Aa, FITC-MSN-treated cells exhibited weak green fluorescence of particles. In contrast, notable green fluorescence was captured for the FITC-HA-MSN group, the intensity of which was more than twice that of FITC-MSNs according to ImageJ data (Figure 5B). Remarkably, Raw264.7 cells treated with free HA (10 mg/mL) before FITC-HA-MSNs treatment showed very weak green fluorescence since free HA blocked the CD 44 receptors on macrophages and therefore hindered the cellular recognition and internalization of HA-MSN. In contrast, particles showed no selective targeting to cells without LPS activation (Figure 5Ab) and no significant difference in the cellular uptake was observed (Figure 5B). Moreover, limited cellular uptake was observed with HUVECs with or without LPS activation and no inhibitory effect on internalization was found in the presence of excessive free HA (Figure S7). Therefore, the above data demonstrated that HA-MSN can selectively target inflammatory macrophages.

Furthermore, intracellular drug release behavior was visualized by loading a lipophilic dye Nile red (NR) instead of SIM into HA-MSN, which exhibited weak fluorescence in aqueous solution but strong red fluorescence in contact with intracellular lipids [45]. The as-obtained NR@HA-MSN was incubated with LPS and oxLDL-treated RAW 264.7 cells to mimic the particle internalization by inflammatory macrophages in the plaque. As a result, the red fluorescence of NR increased in a time-dependent manner both in LPS and oxLDL-treated RAW 264.7 cells 1, 2, 4, and 8 h after co-incubation, indicating that NR was released intracellularly in a sustained manner (Figure S8A,B). Overall, the efficient cellular uptake and sustained cargo release in inflammatory macrophages suggested that HA-MSN may serve as a promising targeted delivery platform for atherosclerosis management.

### 3.7. In Vitro Anti-Atherosclerosis Effects

#### 3.7.1. Downregulating Effects on Inflammation

**Figure 6:**
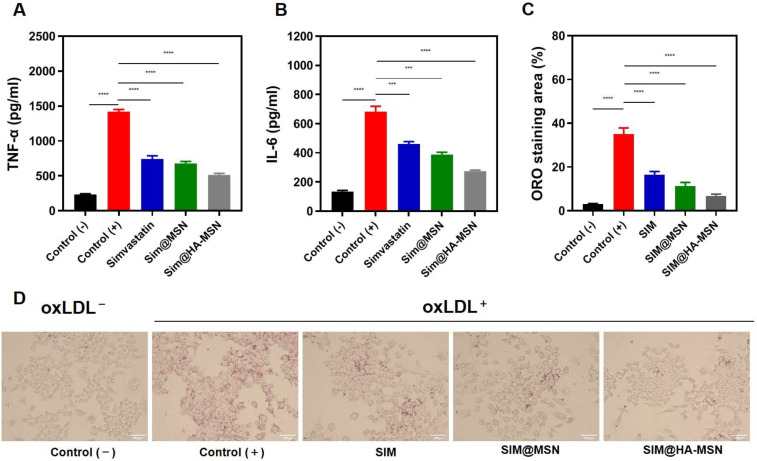
Quantification of proinflammatory cytokines (**A**) TNF-α and (**B**) IL-6 secreted by Raw264.7 cells after different treatments at equivalent SIM concentrations for 24 h (*n* = 3). (**C**) Quantification of oil red O staining areas and (**D**) oil red O staining optical microscopy images of Ox-LDL-induced foam cell formation after various treatments at equivalent SIM concentrations for 24 h (*n* = 3). Variance among groups was determined by one-way ANOVA with Tukey post-hoc test (*** *p* < 0.001, **** *p* < 0.0001).

Proinflammatory cytokines are detrimental factors in the pathogenesis of atherosclerosis [46]. Therefore, typical pro-inflammatory cytokines, namely TNF-α and IL-6 secreted by RAW264.7 cells after different treatments, were quantified by ELISA. As manifested in Figure 6A, levels of TNF-α and IL-6 were significantly attenuated after SIM, SIM@MSN, and SIM@HA-MSN treatment compared with the control group stimulated with LPS only. Strikingly, SIM@HA-MSN treatment led to the lowest level of TNF-α and IL-6 at 514.12 and 273.62 pg/mL, respectively, which were ~70% that of the SIM and SIM@MSN groups (Figure 4C). The potent anti-inflammatory effect of SIM@HA-MSN probably resulted from enhanced internalization.

#### 3.7.2. Inhibitory Effects on Foam Cell Formation

During the pathogenesis of atherosclerosis, oxidized low-density lipoproteins (Ox-LDLs) in the tunica intima can induce the subendothelial infiltration of macrophages and stimulate their differentiation into foam cells that finally contribute to plaque formation [47]. Hence, we evaluated the inhibitory effect of SIM@HA-MSN on senescent foamy macrophage formation induced by ox-LDL (Figure 6C,D). As demonstrated by the quantitative results in Figure 6C, the oil red O (ORO) area of positive cells sharply increased to 35.10 ± 2.78%, indicating a strong foaming effect of ox-LDL on macrophages. Encouragingly, the staining area was robustly reduced after SIM, SIM@MSN, and SIM@HA-MSN treatments. The inhibitory effect of SIM@HA-MSN was the most remarkable, in which the staining area was decreased to 6.77 ± 0.85%, which was around one fifth that in the positive control group. These results confirmed the potent inhibitory effect of SIM@HA-MSN on macrophage foaming, which would potentially contribute to the clearance of the foam cells in the plaque.

### 3.8. Circulating Test In Vivo

**Figure 7:**
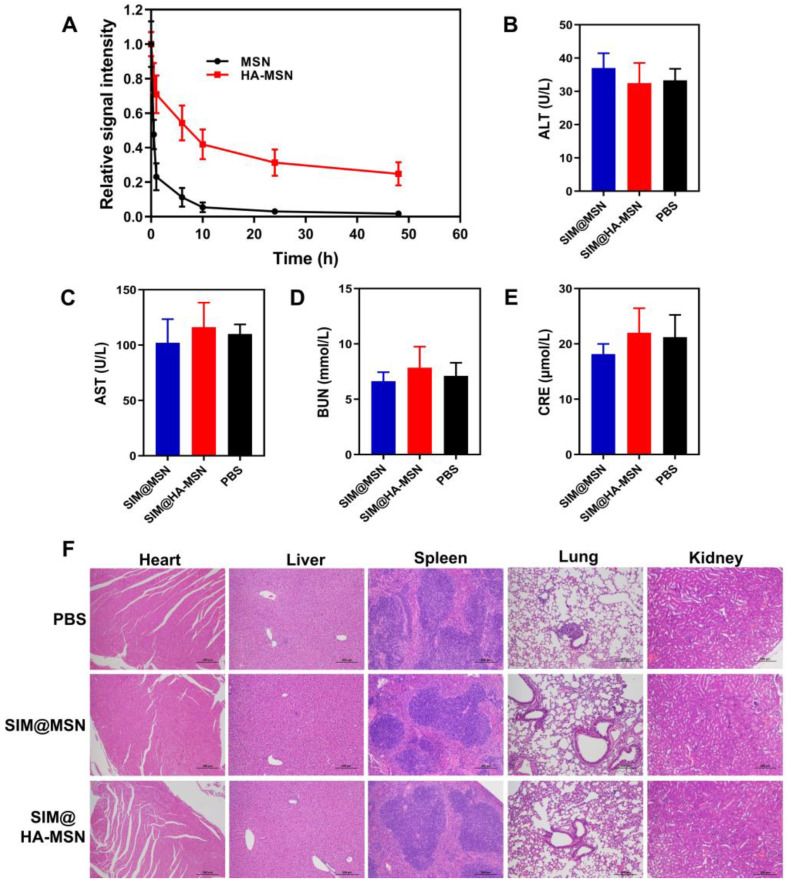
(**A**) Relative fluorescence intensity of FITC-labeled MSN and HA-MSN in blood over 48 h (*n* = 6). (**B**–**E**) Serological biochemistry results of mice treated with SIM@MSN and SIM@HA-MSN at equivalent SIM concentrations for 4 weeks (*n* = 6). (**F**) Representative images of H&E-stained sections of major organs from mice after different treatments for 4 weeks (scale bar = 100 µm).

To assess whether HA-MSN has a long-circulating life, we studied the in vivo fate of FITC-labeled HA-MSN and MSN in C57BL/6 mice. After intravenous injection, the residual content of the nanosystems was evaluated by measuring the relative signal intensity of the blood collected over 48 h using fluorescence spectroscopy. The results showed that HA-MSNs significantly prolonged the retention time in vivo compared with bare MSNs, which were almost eliminated 10 h after injection. Remarkably, about 37% of HA-MSNs remained in the blood 48 h after injection, while the fluorescence intensity of MSNs could hardly be detected (Figure 7A). Thus, HA-MSN can effectively prolong blood circulation time, potentially enhancing the duration of SIM and its ability to target atherosclerotic plaques.

### 3.9. Biosafety Evaluations In Vivo

MSNs are extensively investigated in the field of drug delivery and biomedical engineering, but they are rarely reported for CVD treatment due to their potential toxicity. Herein, liver function, pathological changes of major organs, organ index, and body weights were monitored and evaluated after repetitive injections of SIM@HA-MSN at a therapeutic dose of SIM (15 mg/kg) for one month, which was frequently adopted for SIM-loaded nanotherapeutics to achieve desirable anti-atherosclerosis efficacy according to previous reports [48,49]. SIM@MSN was investigated at the same time as both nanomaterials and the fabrication process could potentially bring in toxicity in vivo. As shown in Figure 7B–E, serum biochemistry analysis showed normal levels of creatinine (CRE), aspartate aminotransferase (AST), alanine aminotransferase (ALT), and urea nitrogen (BUN) levels in the SIM@HA-MSN and SIM@MSN groups, suggesting that the functions of liver and kidney were well preserved after one month of treatment. In parallel, no obvious pathological changes or injuries were observed from H&E-stained sections of major organs including heart, liver, spleen, lung, and kidney derived from mice after treatment with SIM@MSN or SIM@HA-MSN (Figure 7F) compared with the PBS group. Moreover, the organ index and body weights of the mice were also comparable to that of the PBS group, further confirming their biosafety in vivo (Figures S9 and S10). Therefore, the absence of in vivo toxicity or side effects after intravenous administration of therapeutic doses for a month implicated that SIM@HA-MSN can be a safe candidate for atherosclerosis therapy.

## 4. Conclusions

In summary, we achieved successful functionalization of MSNs with HA through two-step conjugations while maintaining high loading efficiency (>20%) of a potent lipid-lowering drug (SIM) for the treatment of atherosclerosis (AS). HA conjugation efficiently reduced the premature leakage of the drug and therefore can potentially assure the enrichment of the drug at the lesion by CD 44 targeting and HAase-triggered drug release in the plaque microenvironment. Interestingly, the as-constructed SIM@HA-MSN exerts significantly stronger capability in attenuating the secretion of proinflammatory cytokines TNF-α and IL-6 and preventing the foaming of macrophages than free SIM and SIM@MSN, probably due to CD 44-mediated enhancements in cellular recognition and internalization. Moreover, HA coating also enhanced the cyto/hemo compatibility and significantly prolonged the plasma retention time of MSN, which can be particularly advantageous in treating vascular disorders, including atherosclerotic plaques. In vivo studies further confirmed its biosafety with well-preserved liver and kidney functions and the absence of pathological changes in major organs. All in all, the as-established SIM@HA-MSN can potentially serve as a safe and effective candidate to combat atherosclerosis due to the ease of fabrication, controlled drug delivery, robust anti-inflammation and anti-foaming efficacy, potent targeting ability, and good biocompatibility. Based on these encouraging data, the targetability and anti-AS effects will be further investigated in depth in an apolipoprotein E-knockout (apoE−/−) mouse model of atherosclerosis.

## Figures and Tables

**Table 1:** BJH and BET analysis of MSN-based systems.

Sample	BET Surface Area (m^2^/g)	Pore Volume (cm^3^/g)	Pore Diameter (nm)
**MSN**	1059.39	1.0316	2.8
**PEI-MSN**	370.79	0.5136	2.2
**HA-MSN**	96.92	0.2661	—
**SIM@HA-MSN**	27.64	0.1866	—

## Data Availability

Data is provided in this article or the Supplementary Materials.

## Supplementary Materials

The following supporting information can be downloaded at: https://www.mdpi.com/article/10.3390/pharmaceutics14061265/s1, Figure S1: Loading efficiency and encapsulation efficiency of SIM by APTES modified MSN (MSN-NH_2_) through drug impregnation method, in which SIM and MSN-NH_2_ were co-immersed in dichloromethane and stirred for 24 h before collection and washing by centrifugation (*n* = 3). Figure S2: (A) Size distribution of MSN, HA-MSN and SIM@HA-MSN determined by DLS. (B) Stability of HA-MSN and SIM@HA-MSN in PBS (10 mM, pH = 7.4) monitored by DLS over 1 week. Figure S3: Impact of solvent type on loading efficiency and encapsulation efficiency of SIM by drug impregnation method (*n* = 3). Figure S4: Impact of stirring time on loading efficiency and encapsulation efficiency of SIM by drug impregnation method (*n* = 3). Figure S5: Impact of loading sequence on loading efficiency and encapsulation efficiency of SIM by drug impregnation method in which SIM was either loaded in MSNs before (drug loading first) or after (drug loading finally) surface modifications (*n* = 3). Figure S6: Cytocompatibility of particles on HUVECs after co-incubation for 24 h (*n* = 3). Figure S7: (A) Confocal laser microscope images of HUVECs cells treated with FITC labeled HA-MSN, MSN, and free HA + FITC-HA-MSN for 2 h with (a) or without (b) LPS activation. (B) Mean fluorescence intensity of FITC-HA-MSN, FITC-MSN, and free HA + FITC-HA-MSN after cellular uptake (*n* = 3). Figure S8: (A) Confocal laser microscope images of LPS- and oxLDL-treated RAW 264.7 cells co-incubated with NR@HA-MSN for 1 h, 2 h, 4 h, 8 h. (B) Corresponding mean fluorescence intensity of dye after cellular internalization (*n* = 3). Figure S9: The organ index of mice after treatment with PBS, SIM@MSN, and SIM@HA-MSN for 4 weeks (%, *n* = 6). Figure S10: Body weights of mice treated with SIM@MSN, SIM@HA-MSN, and PBS for 4 weeks (*n* = 6).
