# Supplementary material for: Hyaluronic Acid-Functionalized Mesoporous Silica Nanoparticles Loading Simvastatin for Targeted Therapy of Atherosclerosis
Source: Pharmaceutics. 2022 Jun 14;14(6):1265. doi: 10.3390/pharmaceutics14061265 (PMC9227583; doi:10.3390/pharmaceutics14061265)
Supplement: Supplementary file 1 [file pharmaceutics-14-01265-s001.zip › pharmaceutics-1751145-supplementary.pdf]

Supplementary information

Hyaluronic Acid-functionalized Mesoporous Silica Nanoparticles Loading Simvastatin for Targeted Therapy of Atherosclerosis

Kechen Song [1,†], Zhuang Tang [1,†], Zhiling Song [1], Shiyu Meng [1], Xiaoxue Yang [1], Hui Guo [2,*], Yizhun Zhu [1] and Xiaolin Wang [1,*]

1 School of Pharmacy and State Key Laboratory of Quality Research in Chinese Medicine, Macau University of Science and Technology, Taipa 999078, Macao, China

2 School of Chemical Engineering and Technology, Sun Yat-sen University, Zhuhai 519082, China

E-mail:

xilwang@must.edu.mo (X. Wang)

guoh37@mail.sysu.edu.cn (H. Guo)

[†] Authors contributed equally to this work.

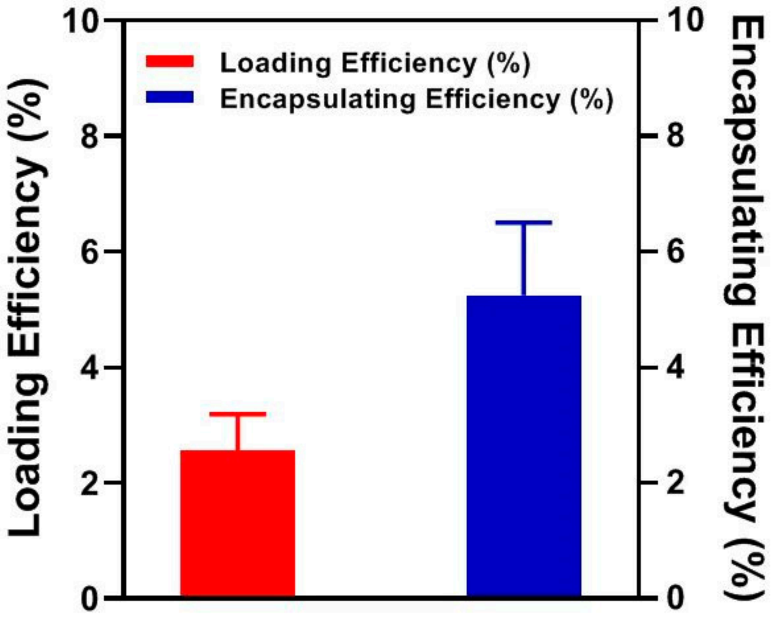

Figure S1 Loading efficiency and encapsulation efficiency of SIM by APTES modified MSN ($MSN-NH_2$) through drug impregnation method, in which SIM and $MSN-NH_2$ were co-immersed in dichloromethane and stirred for 24 h before collection and washing by centrifugation (n = 3).

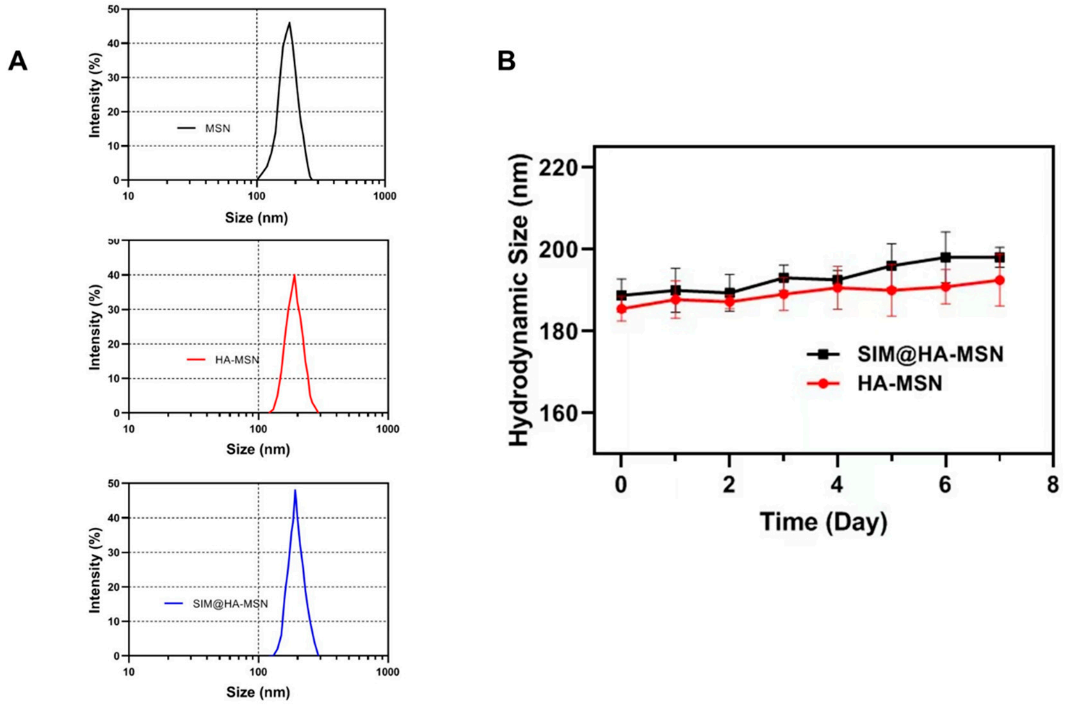

Figure S2 (A) Size distribution of MSN, HA-MSN and SIM@HA-MSN determined by DLS. **(B)** Stability of HA-MSN and SIM@HA-MSN in PBS (10 mM, pH = 7.4) monitored by DLS over 1 week.

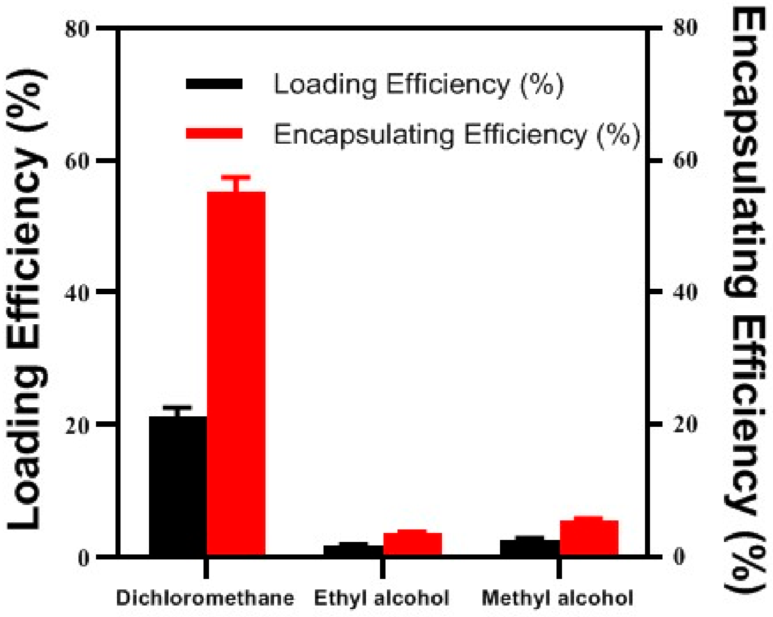

Figure S3 Impact of solvent type on loading efficiency and encapsulation efficiency of SIM by drug impregnation method (n = 3).

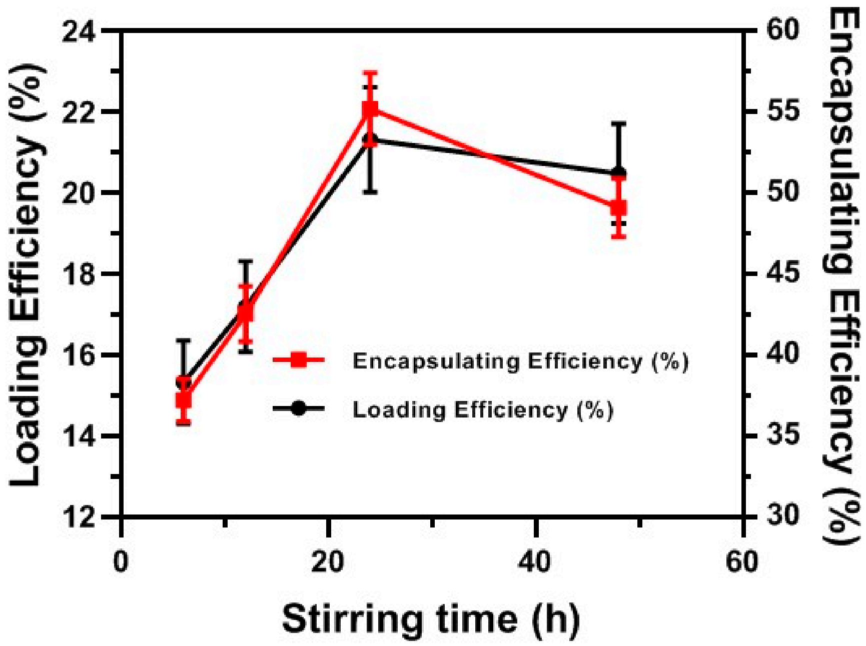

Figure S4 Impact of stirring time on loading efficiency and encapsulation efficiency of SIM by drug impregnation method (n = 3).

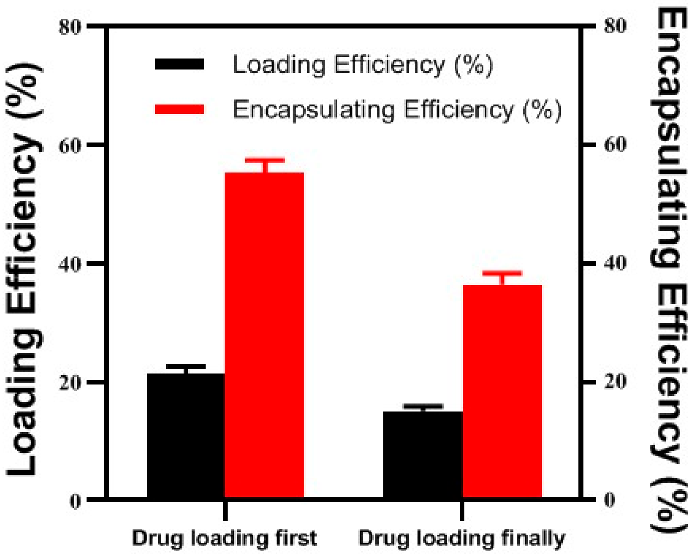

Figure S5 Impact of loading sequence on loading efficiency and encapsulation efficiency of SIM by drug impregnation method in which SIM was either loaded in MSNs before (drug loading first) or after (drug loading finally) surface modifications (n = 3).

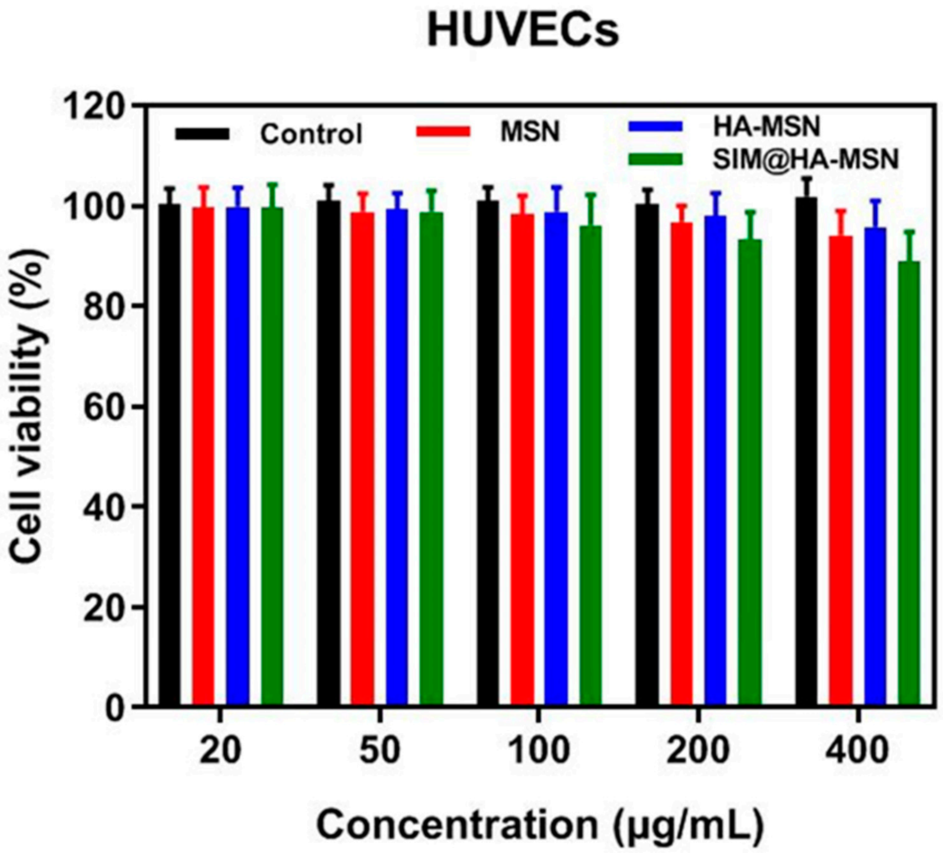

Figure S6 Cytocompatibility of particles on HUVECs after co-incubation for 24 h (n = 3).

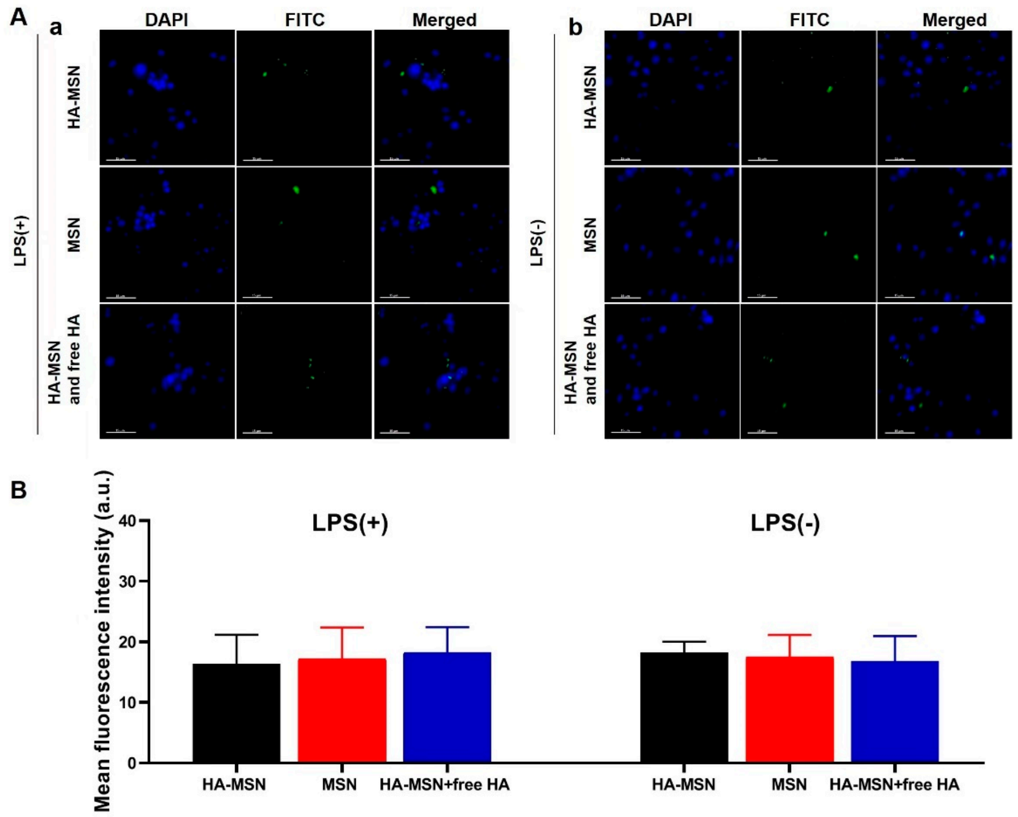

Figure S7 (A) Confocal laser microscope images of HUVECs cells treated with FITC labeled HA-MSN, MSN, and free HA + FITC-HA-MSN for 2 h with (a) or without (b) LPS activation. (B) Mean fluorescence intensity of FITC-HA-MSN, FITC-MSN, and free HA + FITC-HA-MSN after cellular uptake (n = 3).

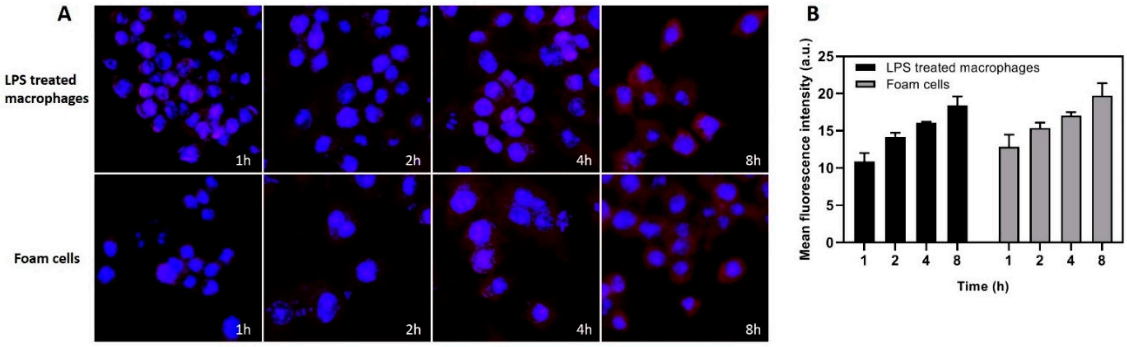

Figure S8 (A) Confocal laser microscope images of LPS- and oxLDL-treated RAW 264.7 cells co-incubated with NR@HA-MSN for 1 h, 2 h, 4 h, 8 h. (B) Corresponding mean fluorescence intensity of dye after cellular internalization (n = 3).

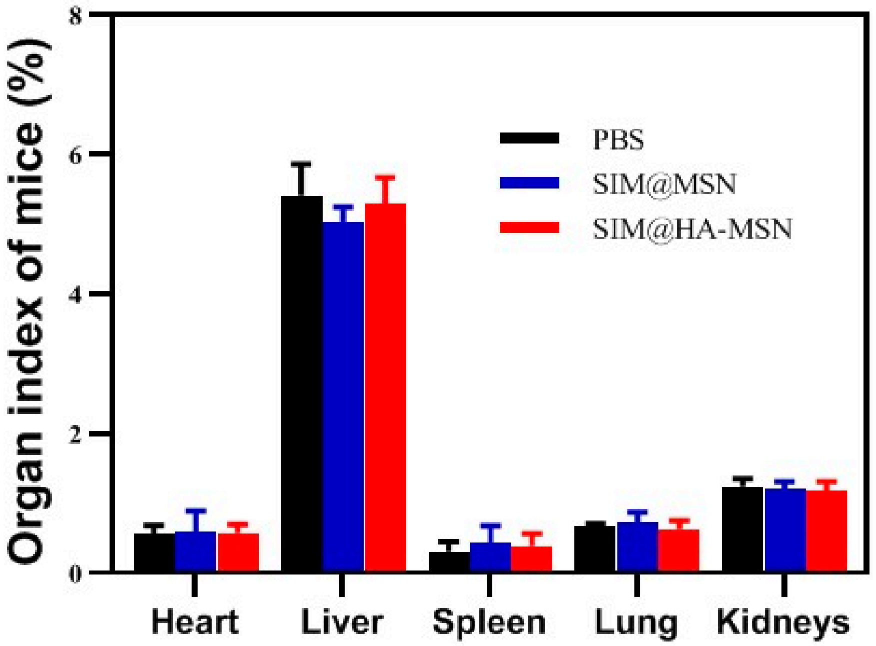

Figure S9 The organ index of mice after treatment with PBS, SIM@MSN, and SIM@HA-MSN for 4 weeks (%, n = 6).

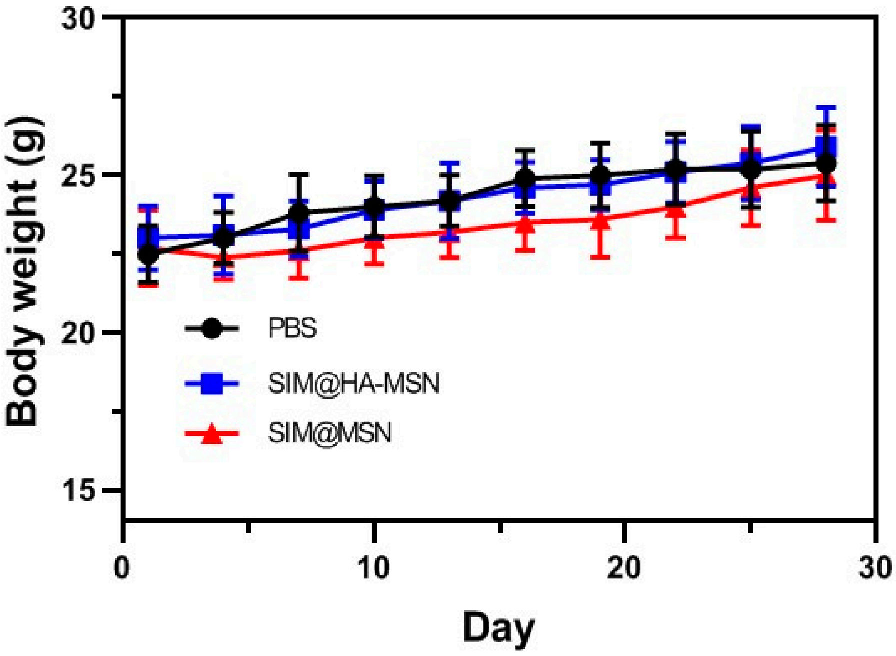

Figure S10 Body weights of mice treated with SIM@MSN, SIM@HA-MSN, and PBS for 4 weeks (n = 6).
